# Clinical Overview of Obesity and Diabetes Mellitus as Risk Factors for Atrial Fibrillation and Sudden Cardiac Death

**DOI:** 10.3389/fphys.2018.01847

**Published:** 2019-01-07

**Authors:** Edwin A. Homan, Michael V. Reyes, Kathleen T. Hickey, John P. Morrow

**Affiliations:** Columbia University Medical Center, New York, NY, United States

**Keywords:** atrial fibrillation, obesity-complications, sudden cardiac death (SCD), diabetes mellitus, arrhythmia (heart rhythm disorders)

## Abstract

The epidemics of obesity and diabetes mellitus are associated with an increased incidence of both atrial fibrillation (AF), the most common sustained arrhythmia in adults, and sudden cardiac death (SCD). Obesity and DM are known to have adverse effects on cardiac structure and function. The pathologic mechanisms are thought to involve cardiac tissue remodeling, metabolic dysregulation, inflammation, and oxidative stress. Clinical data suggest that left atrial size, epicardial fat pad thickness, and other modifiable risk factors such as hypertension, glycemic control, and obstructive sleep apnea may mediate the association with AF. Data from human atrial tissue biopsies demonstrate alterations in atrial lipid content and evidence of mitochondrial dysfunction. With respect to ventricular arrhythmias, abnormalities such as long QT syndrome, frequent premature ventricular contractions, and left ventricular hypertrophy with diastolic dysfunction are commonly observed in obese and diabetic humans. The increased risk of SCD in this population may also be related to excessive cardiac lipid deposition and insulin resistance. While nutritional interventions have had limited success, perhaps due to poor long-term compliance, weight loss and improved cardiorespiratory fitness may reduce the frequency and severity of AF.

## Introduction

Atrial fibrillation (AF) is the most common cardiac arrhythmia in adults and is characterized by chaotic atrial electrical activity leading to irregular heart rhythm. AF is a chronic illness associated with numerous debilitating adverse outcomes including heart failure, dementia, and stroke from embolic events. In addition, AF symptoms such as palpitations, fatigue, and anxiety can further decrease quality of life. It is estimated that 2.7–6.1 million Americans are living with AF and this is expected to grow to 20 million by 2030 owing to an aging population (Benjamin et al., 2018). The cost of caring for patients with AF is a significant economic burden (Patel et al., 2014). AF leads to significant morbidity and mortality despite contemporary interventions (Benjamin et al., 1998).

AF is typically a progressive disease that starts with short episodes (paroxysmal AF) lasting < 7 days. Gradually, AF becomes more frequent and longer lasting in most patients, ultimately progressing to sustained or permanent AF (Lau et al., 2017). It is clinically managed through pharmacologic heart rate control with beta-blockers, calcium-channel blockers, and other antiarrhythmic medications (Van Gelder et al., 2016). A rhythm control strategy can be attempted to return the patient to sinus rhythm through pharmacologic or electrical cardioversion, or increasingly via intracardiac catheter ablation (Piccini and Fauchier, 2016). Incident AF usually refers to a new diagnosis of AF based on billing codes, insurance claims data, hospital discharge diagnosis, or single in-office electrocardiogram (ECG). Recurrent AF refers to one or more episodes of AF occurring after a cardioversion attempt.

Predisposition to AF appears to also be influenced by polygenic risk factors, though their clinical utility is not clear at this time (Muse et al., 2018; Nielsen et al., 2018; Weng et al., 2018). Trends from long-term longitudinal cohort studies in the United States have also found increasing prevalence of AF though AF incidence has been stable (Schnabel et al., 2015). Several traditional cardiovascular risk factors predict AF, including advanced age and male sex, and modifiable risk factors include heart failure, hypertension, and body mass index (BMI) (Staerk et al., 2017). BMI is defined as body mass in kilograms divided by the square of height in meters (normal, overweight and obese categories are 18.5–24.9, 25–29.9, and ≥ 30 kg/m^2^, respectively). A small surgical case series in 1988 first observed that obesity was a predictor of cardiac perioperative AF (Sumeray et al., 1988), encouraging further research in this area.

## Early Data on Obesity and AF: The Framingham Heart Study

The Framingham Heart Study (FHS) is an ongoing multigenerational, community-based cohort study of cardiovascular disease (CVD), the first of its kind, that started in 1948. The original cohort consisted of 5,209 residents of Framingham, Massachusetts who were examined every 2–4 years, and it was initially planned as a 20-year study to identify risk factors for coronary artery disease (Higgins, 1984). The study was continued to include multiple generations of Framingham residents after the observation of familial inheritance of risk factors. Its scope has expanded to include new biomarkers, new cardiovascular imaging techniques, genomics, proteomics, metabolomics, and social networks. Over the past six decades, seminal insights from the FHS have shaped public health guidelines for the prevention of CVD (Tsao and Vasan, 2015).

The association between metabolic disease and AF was detected in a 1994 study of 4,700 subjects from the FHS original cohort (Benjamin et al., 1994). Over 38 years of follow-up with biennial examinations, 560 subjects developed AF. Presence of hypertension and DM at a given examination, but not obesity, were each associated with incident AF based on multivariate analysis. ECG evidence of left ventricular hypertrophy was also a risk factor for AF, though congestive heart failure was more strongly associated with AF overall. While obesity was not significantly associated with AF in this study, a prior analysis of the FHS cohort had demonstrated that obesity and left ventricular mass were closely correlated (Lauer et al., 1991). Obesity was known to occur in conjunction with many of the AF risk factors identified to that point, but it was not known whether obesity itself was an independent risk factor. Importantly, in this 1994 study, risk factors were tabulated at each examination rather than being prospectively followed after the intake examination. As such, the study only evaluated contemporaneous risk factors at the time of AF incidence rather than conditions that were predictive of future risk of AF.

A 2004 analysis of the FHS data looking specifically at obesity instead used baseline characteristics to predict incident AF during 14 years of follow-up (Wang et al., 2004). Data were gathered from 5,282 participants from both the original and the offspring FHS cohorts who did not have AF at baseline. Strikingly, BMI predicted AF and there was no threshold effect. Each single-unit increment of BMI above the normal range was associated with a 4% increased risk of AF, even after adjustment for other traditional cardiovascular risk factors including diabetes mellitus (DM) and hypertension. Overall, the age-adjusted hazard ratio (HR) was 1.52 for obese men and 1.46 for obese women, and even overweight individuals were at significantly higher risk of AF. The retrospective cohort design of this study likely accounts for differing conclusions in obesity as a risk factor. Importantly, the freedom-from-AF curves with respect to baseline obesity did not diverge until 8–10 years after the initial examination. Adjustment for left atrial diameter markedly attenuated the association between obesity and AF, suggesting that atrial structural remodeling may partially mediate the association between obesity and AF.

A clinical risk score for future AF incidence was subsequently developed based on the FHS data over 10-year follow-up during which 10% of participants developed AF (Schnabel et al., 2009). In this predominantly Caucasian, middle-aged to elderly cohort, the clinical variables associated with AF were age, sex, BMI ≥ 30, systolic blood pressure, treatment for hypertension, PR interval, clinically significant cardiac murmur, and heart failure. Age and early-onset heart failure were the largest contributors to the risk score and obesity was a minor component. DM was not included, as the association was not found in the FHS cohorts with longer follow-up (Fontes et al., 2012). A subsequent analysis validated the risk score in both Caucasian and African-American populations, and obesity remained a significant AF risk factor in the Framingham risk score (Schnabel et al., 2010).

## Obesity and Incident AF

Other large international studies have examined whether obesity is an independent risk factor for incident AF (Table 1). A large Danish study examined the relationship between obesity and diagnosis of AF (Frost et al., 2005). In this population-based prospective cohort study, 47,589 participants aged 50–64 without CVD at enrollment were followed from 1993 to 2001. The authors found that each increment of BMI at baseline was associated with 8% increased risk of AF/AFL in men and 6% increase in women over the mean 5.7-year follow-up. For the category of obesity (BMI ≥ 30), the adjusted HR was 2.35 in men and 1.99 in women.

**Table 1:** Cohort studies examining the association of obesity and incident or recurrent AF.

**References**	**Design (location)**	**Patient population**	**Study size**	**Mean follow-up period**	**Results**
Benjamin et al., 1994 (Framingham)	Prospective cohort (Massachusetts)	Community dwelling adults age 55–94, no AF at baseline	4731	38 years	560 new AF cases; BMI trend to significance. DM (OR 1.4 for men, 1.6 for women), age, HTN, and CHF all associated with AF
Wang et al., 2004 (Framingham)	Prospective cohort (Massachusetts)	Community dwelling adults and offspring, no AF at baseline	5282	13.7 years	Each 1-pt increase in BMI associated with 4% increased risk of AF. For BMI ≥ 30, HR 1.52 and 1.46 for men and women, respectively.
Frost et al., 2005	Nationwide prospective cohort (Denmark)	Population-based, age 50–64, no CVD	47,589	5.7 years	Each 1-pt increase in BMI associated with 8% increased risk of AF in men, 6% in women. For BMI ≥ 30, HR 2.35 for men, 1.99 for women.
Umetani et al., 2007	Single-center prospective cohort (Japan)	Initially hospitalized adults in sinus rhythm, no active CVD	592	20 months	Reported HR 3.0 for AF incidence in BMI ≥ 25, which persisted after adjustment for age or LA size.
Watanabe et al., 2008	Prospective cohort (Japan)	Community dwelling adults recruited from annual check-up visits	28,449	4.5 years	HR 1.64 for BMI ≥ 25 and AF incidence. AF also associated with hypertension, dyslipidemia, and hyperglycemia.
Guglin et al., 2011 (AFFIRM)	Subset analysis of a multicenter randomized trial (North America)	Chronic AF, age >65, and high stroke risk	2,518	3.5 years	Higher risk of cardioversion (OR 1.09) or presence of AF on follow-up (OR 1.10) for BMI increase of 5. Effect mediated by LA size.
Dublin et al., 2006	Population-based case-control study (Washington State)	Inpatient or outpatient, aged 30–84 with new AF diagnosis and matched controls	425 AF cases, 707 controls	6 months	Higher BMI increased AF risk by 3% per 1 pt BMI. AF burden also directly related to BMI.
Thacker et al., 2013	Population-based inception cohort study (Washington State)	Adults aged 30–84 with prior episode AF	1,385	5 years	Overall 24% progressed to permanent AF. Compared to normal BMI, obesity increased permanent AF risk (HR 1.35 for BMI 30–34.9)
Tsang et al., 2008	Single-center longitudinal cohort study (Minnesota)	Adults who developed new AF during 1980–2000, then followed passively	3,248	5.1 years	Overall 17% progressed to permanent AF. Compared to normal BMI, obesity increased permanent AF risk (HR 1.54 for BMI 30–34.9). LA volume did not weaken the association
Huxley et al., 2011 (ARIC)	Multicenter longitudinal cohort study (United States)	Community-dwelling adults aged 45–64 at time of enrollment	14,598	17.1 years	AF incidence higher in white men vs. black women (7.4 vs. 3.7 per 1000 person-years). AF risk driven primarily by hypertension. Overall attributable risk to elevated BMI ≥ 30 was 13%.
Rodriguez et al., 2015 (MESA)	Multicenter longitudinal cohort study (United States)	Multiethnic, gender balanced, age 45–84, without known CVD	6,721	7.3 years	Lower rate of AF in non-white groups (incidence rate ratios 0.35, 0.51, 0.54 in Asians, Hispanics and non-Hispanic blacks, respectively). BMI correlated with AF in Hispanics only.
Jensen et al., 2013 (Cardiovascular Health Study)	Multicenter longitudinal cohort study (United States)	Multiracial, age > 65, no known AF at baseline	5,685 (911 blacks)	11.2 years	AF risk was 25% lower in blacks overall, and 45% after adjustment for CVD risk factors. BMI was stronger predictor of AF in blacks than whites.
Austin et al., 2018 (Jackson Heart Study)	Community-based longitudinal cohort study (Mississippi)	African-Americans age 35–84, recruited starting in 2000.	5,306	8.5 years	Obesity was independent predictor of AF (HR 1.24 for each additional 15 kg).
Rodriguez et al., 2016 (Women's Health Initiative)	Multicenter longitudinal cohort study (United States)	Multiethnic women age 50–79, post-menopausal	134,936	13.7 years	Despite higher RF burden in non-white groups, AF incidence was lower in these groups compared to whites (0.4–0.7% vs. 1.2%).

*AF, atrial fibrillation; BMI, body mass index (kg/m^2^); CHF, congestive heart failure; CVD, cardiovascular disease; HR, hazard ratio; HTN, hypertension; LA, left atrium; OR, odds ratio; RF, risk factors*.

A smaller prospective single-center study in Japan of 592 consecutive hospitalized patients in normal sinus rhythm on admission found that BMI ≥ 25 was a significant risk factor for development of AF or AFL during an average 20-month follow-up (Umetani et al., 2007). Other aspects of metabolic syndrome such as DM and dyslipidemia were not significantly associated with AF. Strikingly, obesity was associated with AF even after adjustment for age or increased left atrial diameter. Patients with structural heart disease (including reduced ejection fraction) or chronic AF were excluded, and most had been admitted for evaluation of ischemic cardiac disease.

A large prospective cohort study in Japan included 28,449 participants without baseline AF (Watanabe et al., 2008). Notably, participants were community dwellers who were followed over a mean 4.5 years. As before, obesity (defined as BMI ≥ 25 in this East Asian population) was associated with increased risk of AF (adjusted HR 1.64). However, in contrast to existing studies in Western populations at the time, increased AF incidence was also associated with other components of the metabolic syndrome, including elevated blood pressure, low high-density cholesterol, and impaired fasting glucose. The location of the excess adipose tissue may be important. Some studies indicate that epicardial fat is a better predictor of AF than overall adiposity (Wong et al., 2011, 2016).

## Obesity and AF Recurrence

The 2002 AFFIRM trial was a landmark multicenter randomized trial that compared rhythm control and rate control strategies in 4,060 subjects with baseline AF. A subset analysis of 2,518 subjects with BMI data found that obesity, age, hypertension, and left atrial size were independently associated with AF recurrence as measured by number of attempted cardioversions and AF burden (Guglin et al., 2011). In multivariate analysis, only left atrial size was associated with both AF recurrence and AF burden, though BMI strongly correlated with left atrial size in this group.

A population-based case-control study of 425 subjects with new-onset AF found obesity to be more closely linked to persistent AF than to paroxysmal AF (Dublin et al., 2006). The subjects were drawn from Washington State and were predominantly Caucasian. Each increment of BMI was associated with 3% high AF risk overall, and there was a progressively stronger association with more sustained AF. That is, for each BMI increment, there was 7% higher risk of sustained AF (≥6 months), 3% for intermittent AF (≥8 days or recurrent), and 1% for transitory AF (< 8 days). DM appeared to have a small but measurable role in mediating this association.

In a 2013 population-based cohort study from the same Washington State health care system, 1,385 participants with newly diagnosed AF in 2001–2004 were monitored for progression to permanent AF (Thacker et al., 2013). Only subjects whose initial AF terminated within 6 months were included, thus establishing that they did not have permanent AF at baseline. In multivariate analysis, increasing BMI was independently associated with progression to permanent AF, but DM and hypertension were not.

A longitudinal cohort study identified 3,248 Minnesota residents who developed paroxysmal AF and followed them for up to 20 years (Tsang et al., 2008). Over 5.1 years of follow-up, 17% of subjects progressed to permanent AF. Compared to normal BMI, obesity (BMI 30–34.9) and severe obesity (BMI ≥35) were associated with a 54 and 89% increased risk of progression from paroxysmal to permanent AF, respectively. In a subgroup with echocardiographic data, left atrial volume did not weaken the association between BMI category and progression to permanent AF.

In summary, several small-to-moderately sized longitudinal cohort studies suggested that obesity is a risk factor for permanent AF and AF recurrence, possibly mediated in part by left atrial volume. Comparison between studies is hampered by variable definitions of paroxysmal AF used and methods of assessment of presence of AF.

## Obesity and AF Incidence in Multiethnic Groups

A lack of ethnic diversity within some of the early cohort studies limited their generalizability, prompting more studies with ethnically diverse populations. The Atherosclerosis Risk In Communities (ARIC) community-based cohort study recruited 14,598 white and black middle-aged participants from 1987 to 1989 in the eastern United States with mean follow-up period of 17 years (Huxley et al., 2011). The study attempted to establish the fraction of AF incidence that was attributable to specific risk factors of blood pressure, BMI, DM, smoking, and history of cardiac disease. Age-adjusted incidence of AF was highest in white men and lowest in black women (7.4 and 3.7 per 1,000 person-years, respectively). Overall, 56% of AF incidence was attributable to having at least one borderline or elevated risk factor, driven primarily by hypertension (22%), and elevated BMI (13%), with DM as only a minor contributor (3%). A follow-up study found that prevalence of obesity peaked in the decade prior to AF incidence, which was not seen with the other risk factors (Norby et al., 2016).

To expand the scope of these findings to Hispanic and Asian populations, the Multi-Ethnic Study of Atherosclerosis community-based cohort study monitored for incident AF in 6,663 older subjects using hospital discharge codes (Rodriguez et al., 2015). Overall, despite a more favorable risk factor profile in non-Hispanic whites, AF incidence rates were significantly lower in Asian-Americans, Hispanics, or non-Hispanic blacks; incident rate ratios were 0.35, 0.51, and 0.54, respectively. The risk of AF incidence attributable to hypertension was significantly higher in the non-white groups. Interestingly, BMI was closely related to AF incidence in the Hispanic group, but not in the other groups.

The Cardiovascular Health Study recruited 5,685 older adults >65 years of age, of which 911 were black, and AF incidence was tracked over an average 11.2 years (Jensen et al., 2013). Overall AF risk was 25% lower in black subjects after adjustment for age and sex alone, and 45% lower after adjustment for all considered risk factors. Nevertheless, elevated BMI was a stronger predictor of AF in blacks than whites.

Attention focused exclusively on an African-American population in the Jackson Heart study, which recruited 5,306 black subjects from 2000 to 2004 (Knight and Sumner, 2011). Over an average 8.5 years follow-up, 242 cases of incident AF were identified, with similar incidence rates to other cohort studies (Austin et al., 2018). High body weight was associated with increased risk of incident AF (HR 1.23 per 15 kg), though BMI was not specifically measured. Nevertheless, elevated bodyweight remained a strong predictor of incident AF in their multivariable model. Consistent with the ARIC study, while DM was initially associated with AF in the age- and sex-adjusted model, it was not in the multivariable model.

The ethnically diverse Women's Health Initiative examined a large population cohort study of 134,936 postmenopausal women free of AF at baseline (Rodriguez et al., 2016). Despite a higher burden of hypertension, DM, and obesity, annual AF incidence was lower among non-whites (0.7, 0.4, and 0.4% for non-Hispanic black, Hispanic, and Asian-American participants, respectively), compared with 1.2% for non-Hispanic whites. Obesity and DM were significant risk factors for AF incidence across the ethnic groups.

In four studies with significant non-white populations, there were lower overall AF incidence rates in the non-white subgroups, but obesity remained a significant risk factor for AF incidence.

## Obstructive Sleep Apnea Partially Mediates AF Risk in Obesity

Risk of obstructive sleep apnea (OSA), a type of sleep-disordered breathing, increases dramatically with obesity. Occult OSA may be responsible for some pathophysiologic characteristics traditionally attributed to obesity. OSA causes intermittent nocturnal hypoxia, retention of carbon dioxide, and sympathetic activation. Observational studies in the late 1990s initially found that prevalence of AF was higher in patients with sleep-disordered breathing and concomitant heart failure or recent coronary artery bypass surgery (Mooe et al., 1996; Javaheri et al., 1998; Sin et al., 1999). A small prospective study was the first to show that treatment of OSA using nocturnal continuous positive airway pressure (CPAP) decreased AF recurrence after electrical cardioversion (Kanagala et al., 2003).

A cross-sectional study of 151 AF patients undergoing elective electrical cardioversion found a higher prevalence of OSA (49 vs. 32%) compared to general cardiology patient controls (Gami et al., 2004). While obesity was also associated with both AF recurrence and OSA, the association of AF recurrence and OSA (OR 2.19) persisted after control for BMI and other covariables. Researchers later conducted a retrospective cohort study of 3,542 participants without AF who were referred for initial sleep study from 1987 to 2003 (Gami et al., 2007). During mean follow-up of 4.7 years, new-onset AF occurred in 133 subjects. In younger subjects < 65-years-old, obesity and magnitude of nocturnal desaturation were independent predictors of incident AF.

Other studies of recurrent AF after catheter ablation have yielded differing results. In a prospective study of 109 patients undergoing catheter ablation of drug-refractory AF, only BMI was an independent predictor of treatment failure (defined as < 90% reduction in AF burden) at mean 11-month follow-up (Chilukuri et al., 2010). Nevertheless, another prospective study of 153 patients undergoing catheter ablation for AF found appropriate treatment with CPAP for OSA markedly reduced the rate of AF recurrence post-ablation by 60% (Naruse et al., 2013). An ongoing clinical trial in Norway is recruiting 120 participants with moderate-to-severe sleep apnea and paroxysmal AF undergoing ablation, to be randomized to CPAP vs. no CPAP and AF recurrence will be monitored for 6 months with an implantable event monitor.

## Weight loss and Exercise in AF Risk Reduction

Cardiovascular risk factor reduction, including weight loss, blood pressure control, and glycemic control, was shown to reduce AF frequency and symptoms in a small observational study in Australia of obese and overweight patients undergoing catheter ablation of drug-refractory AF (Pathak et al., 2014). However, this study did not determine how much of the benefit was from improved nutrition vs. better hypertension control, smoking cessation, or other factors. Another study of obese and overweight subjects with paroxysmal AF demonstrated that long-term weight loss can decrease AF burden in a dose-dependent fashion (Pathak et al., 2015a). Participants were followed for a mean 4 years, and >10% weight loss was associated with better arrhythmia-free survival (86 vs. 39%) and lower antiarrhythmic drug use compared to minimal or no weight loss.

Similar results were also seen in a small trial of 150 Australian adults who were randomized (rather than simply observed) to a supervised weight loss program or self-directed lifestyle measures (Abed et al., 2013a). At 15-month follow-up, the intervention group had significantly greater weight loss (14.3 vs. 3.6 kg), which resulted in reduced AF burden and decreased LA area. In addition, bariatric surgery has been shown to significantly reduce the risk of AF, which supports obesity as a causal factor in AF pathophysiology (Jamaly et al., 2016).

Although exercise is beneficial in the general population, intense exercise can be harmful in certain genetic syndromes, such as arrhythmogenic right ventricular cardiomyopathy (ARVC). About half of these patients develop atrial arrhythmias (Chu et al., 2010). It is worth noting that exercise might not be recommended for individuals with mutations that cause atrial fibrillation and cardiomyopathy. Furthermore, intense endurance exercise appears to increase LA size in elite or recreational athletes and may subsequently predispose to AF (Iskandar et al., 2015; Elliott et al., 2018; Opondo et al., 2018). This is a dose-responsive effect that increases with lifetime hours of participation in endurance athletic activity (Flannery et al., 2017).

Nevertheless, overall cardiorespiratory fitness (CRF) appears independently to provide protection from AF burden in older overweight subjects. An observational study of 308 Australian adults with BMI ≥ 27 initially stratified subjects by low (< 85% predicted), adequate (85–100% predicted), or high CRF (>100% predicted) (Pathak et al., 2015b). All subjects participated in a tailored moderate-intensity exercise program and assessed for AF with 7-day continuous ambulatory ECG monitoring. At mean 4-year follow-up, 12% of the low CRF group remained free of arrhythmia without drugs or ablation, compared to 66% of the high CRF group. Participants who increased their CRF by ≥2 metabolic equivalents showed improvement in LV diastolic dysfunction and reduction of LA size. Each unit increase in CRF was associated with a 13% decrease in risk of AF recurrence, independent of any subsequent weight loss. The disparate conclusions compared to studies in athletes may be related to the lower intensity of exercise and direct measurement of changes in exercise capacity over time.

Another longitudinal study looked at the impact of functional aerobic capacity on AF incidence among 12,043 community-dwelling adults in Minnesota (Hussain et al., 2018). During median follow-up of 14 years, 1,222 people developed AF. Each 10% increase in functional aerobic capacity was associated with 7% decrease in incident AF, adjusted for several clinical variables including BMI. These findings suggest that fitness may offset some of the deleterious effects of fatness on AF risk.

There may be an obesity paradox in AF; that is, obese patients with AF have better outcomes than non-obese patients with AF (Proietti et al., 2017). This may be partly explained by the fact that obese people with AF are younger on average. In the international prospective registry study PREFER in AF, 6,196 adults were stratified into asymptomatic and symptomatic AF based on clinical European Heart Rhythm Association scores (category I vs. categories II–VI) (Bakhai et al., 2016). While bodyweight was slightly higher in symptomatic subjects, there were no differences in prevalence of obesity or DM. Nonetheless it remains possible that obesity promotes AF, but also has some mild protective effect that reduces the risk of stroke. The molecular mechanisms have not been examined. Several ongoing clinical trials in Texas, Germany, and the United Kingdom are currently exploring the direct effect of intensive lifestyle and weight reduction on AF recurrence after catheter ablation.

## Dietary Modification and AF Risk Reduction

Although the risk factors for AF and atherosclerosis overlap, the traditional cholesterol panel does not predict new-onset AF (Schnabel et al., 2009), and other aspects of lipid metabolism may be more important for AF pathophysiology. The effect of polyunsaturated fatty acids (PUFA) on AF risk was recently reviewed and found that PUFA supplementation or fish consumption were largely beneficial, though some studies showed no benefit (Kumar et al., 2012a). A *post hoc* analysis of a clinical trial of the Mediterranean diet intervention showed that increasing olive oil consumption decreases the risk of AF (Martínez-González et al., 2014). Observational studies have shown that the consumption of fish, which is rich in PUFA, is inversely related to incident AF (Mozaffarian et al., 2004). Another study examined serum metabolites in samples from the Cardiovascular Health cohort and found that higher levels of PUFA (typically from fish intake) were associated with lower rate of new-onset AF (Wu et al., 2012). A large Italian population study found that PUFA supplementation reduced 1 year risk of AF in patients hospitalized for acute myocardial infarction (Macchia et al., 2008).

The FORWARD trial of 586 adults with symptomatic paroxysmal AF requiring electrical cardioversion found no significant difference at 1 year between subjects given relatively low-dose PUFA (1g/day) or placebo (though serum PUFA levels were not measured) (Macchia et al., 2013). Nevertheless, other studies have found that PUFA supplementation can indeed prevent AF recurrence. An open-label randomized trial of 178 adults with persistent AF found that fish oil supplementation, with confirmed elevated PUFA serum levels, significantly reduced AF recurrence after electrical cardioversion (38 vs. 77%) (Kumar et al., 2012b). On the whole, these findings, while not consistent across different AF subgroups, indicate that diet can influence the incidence and/or progression of AF and suggests that targeted nutritional intervention could be useful for AF patients.

## Obesity and AF: Summary

The weight of evidence from multiple studies shows that obesity contributes significantly to risk of new-onset AF and AF recurrence. There is significant heterogeneity between studies in how AF incidence was measured and defined, as some studies used chart diagnoses and or in-clinic ECG screening, and some studies used longer term event monitoring. The increased risk of AF in obesity appears to be partially but not completely mediated by increases in left atrial size. The increased AF incidence in male sex may be related to population-level height differences; that is, men are taller on average and height correlates directly with larger atrial size, which can predispose to AF (Alonso et al., 2013). European ancestry appears to be a risk factor for AF incidence and is not attributable to differences in risk factor prevalence (Marcus et al., 2010). Lifestyle modifications, including moderate exercise and possibly PUFA supplementation, can attenuate the risk of AF in obese patients.

## Evidence for the Association of DM and AF Risk

Early evidence from the FHS in 1994 suggested a link between DM and AF (Benjamin et al., 1994). A subsequent 3 year prospective study of 4,844 American older adults found elevated blood glucose to be a risk factor for incident AF (Psaty et al., 1997), and a small Spanish case-control study also found DM to be an independent risk factor for AF with odds ratio (OR) 1.9 (Barriales Alvarez et al., 1999). Not all studies have found a significant association: a prospective study of 7,495 Swedish men originally enrolled for smoking cessation and hypertension management with mean follow-up of 25 years looked at hospitalization for AF as the primary endpoint and found no association between DM and AF, though BMI was a strong contributor (Wilhelmsen et al., 2001).

However, a community-based cross-sectional 2004 study in Sweden of 1,739 adults found increased AF prevalence in diabetic adults with or without hypertension (age and sex-adjusted OR 3.3 and 2.0, respectively, compared to healthy controls) (Ostgren et al., 2004). Interestingly, this association was abrogated in both groups by adjustment for insulin resistance, suggesting metabolic dysregulation plays a major role. In a later registry-based observational study of 83,162 Swedish adults with DM but no baseline AF, BMI/obesity, hypertension and microalbuminuria were strong predictors of AF incidence in DM (Zethelius et al., 2015).

A retrospective analysis looked at inpatient records from 845,748 predominantly male patients admitted to a U.S. Veterans Administration hospital between 1990 and 2000. The study found increased AF prevalence in patients with DM compared to patients with hypertension but no DM (adjusted OR 2.1) (Movahed et al., 2005). The VALUE trial randomized 15,245 high-risk hypertensive adults from 31 countries to valsartan or amlodipine treatment with an average 4.2 years of follow-up (Julius et al., 2004). Similarly to the VA study, subjects who developed new-onset DM during the study, but not those with baseline DM, had higher rate of both new-onset AF (adjusted HR 1.49) and persistent AF (HR 1.87) (Aksnes et al., 2008).

Other studies sought to correlate the level of glycemic control with subsequent risk of AF. A 2010 case-control study of 3,613 adults from Washington state, found that overall adjusted OR of AF incidence was 1.40 for people with DM compared to people without DM (Dublin et al., 2010). Relative risk of AF increased by 4% with each additional year of DM duration and also increased with the level of glycated hemoglobin A1C (HbA1c), a measure of overall glycemic control. The ACCORD trial was conducted in 77 clinical centers across North America and looked at cardiovascular outcomes after randomization of 10,082 diabetic subjects to intensive or standard glycemic control (HbA1c targets < 6.0% or 7.0–7.9%, respectively) (Gerstein et al., 2008). However, an analysis of the study data found no difference in AF incidence between the two study groups, although BMI was again independently associated with AF (Fatemi et al., 2014).

A large registry-based prospective cohort study in Spain that included 262,892 records of hypertensive adults without CVD found that DM was modestly associated with new-onset AF (age-adjusted HR 1.20) and there was a non-significant trend toward increased AF risk with HbA1c ≥ 7.0% (Alves-Cabratosa et al., 2016). DM duration was not associated with AF. In another large national registry-based study from Denmark that included 5 million adults who were followed for up to 16 years, DM was associated with increased risk of new-onset AF, an effect that decreased with age (Pallisgaard et al., 2016). The adjusted incident rate ratio decreased from 2.34 in age 18–39 to 1.52 in age 40–64 to 1.20 in age 65–74 to 0.99 for age ≥75.

This age-related effect may be related to development of other AF risk factors over time, which was addressed by researchers in an analysis from the Women's Health Study consisting of 34,720 female health professionals who were free of CVD or AF at baseline and followed for a median 16.4 years (Schoen et al., 2012). Risk of incident AF in women with DM at baseline carried an HR of 1.95 by simple age-adjusted model, which decreased to 1.87 by multivariable-adjusted model and further decreased to 1.37 when both hypertension and BMI were included in the adjustment. However, when time-updated BMI and hypertension measurements were included in the risk model incident AF for subjects with baseline or new DM, the HR fell to 1.19 and was no longer statistically significant.

A metanalysis of 32 cohort studies involving 10 million participants helped to clarify this key question (Aune et al., 2018). After excluding a large outlier retrospective study, the relative risk of AF in the presence of DM was 1.28 (95% confidence interval, CI 1.22–1.35). The authors conclude that there was a dose-response relationship between elevated blood glucose and risk of AF with relative risk 1.11 per 20 mg/dL rise in fasting blood glucose.

Interestingly, metformin use may independently protect diabetic patients from new-onset AF. A recent study of 645,000 diabetic adults from a national registry in Taiwan found lower rates of incident AF in metformin users not using other antihyperglycemics compared to non-users (HR 0.81), possibly by attenuation of oxidative stress and atrial cell myolysis (Chang et al., 2014). Since metformin is a first-line antihyperglycemic medication for DM, this observation may account for the weak association seen at mildly elevated HbA1c values. Preliminary work in canine and human cardiomyocytes has shown that adenosine monophosphate-activated kinase, a cellular energy sensor and purported target of metformin, is activated by atrial fibrillation and metabolic stress (Harada et al., 2015). Further work is needed to clinically correlate this finding.

In summary, the preponderance of clinical evidence suggests that DM is indeed a risk factor for AF with a significant contribution that is independent of co-variables such as BMI or hypertension. This effect may be stronger at younger ages when patients have fewer AF risk factors other than DM.

## Obesity and Diabetes and Risk of Sudden Cardiac Death

Sudden cardiac death (SCD), defined as death attributable to CVD occurring within 1 h of symptom onset, usually arises from life-threatening ventricular tachyarrhythmias leading to hemodynamic instability, shock, and death. While some SCD is due to acute myocardial infarction, obesity is only a weak predictor of coronary artery disease (Schnabel et al., 2009). Therefore the association between obesity and SCD is probably not mediated entirely by excess risk of atherosclerosis and acute myocardial infarction. Given the abrupt time-course and heterogeneity of etiologies, there have been comparatively fewer population-based studies looking at the association between cardiometabolic disease and risk of SCD.

Other work has elucidated the abnormal electrical properties of the heart in obesity. Studies using ECG analysis and continuous ambulatory ECG monitoring have demonstrated that obese humans have increased frequency of ventricular ectopy and greater incidence of abnormally long QT interval (Messerli et al., 1987; Zemva and Zemva, 2000; Ramirez et al., 2011). Ventricular ectopy in the form of frequent premature ventricular contractions are not generally considered harmful, but they are associated with increased risk of ventricular tachyarrhythmias and overall mortality in the general population, even when the overall burden is low (Engel et al., 2007; Hirose et al., 2010). Long QT interval has been shown to be an independent risk factor for cardiovascular mortality (Okin et al., 2004; Watanabe et al., 2007). Furthermore, humans with obesity or DM have increased repolarization dispersion (Wei et al., 1995; Naas et al., 1998; Seyfeli et al., 2006), and QT dispersion predicts SCD in the general population (de Bruyne et al., 1998; Elming et al., 1998; Panikkath et al., 2011).

One of the first descriptions of the association between obesity and SCD comes from the Framingham cohort. In a 1975 study of 4,120 men from both Albany, New York and Framingham, Massachusetts who had been followed for 16 years, the risk of sudden death was 2.4 times higher in obese men than in average weight men (Kannel et al., 1975). A 1983 analysis of data from 5,209 participants in the FHS original cohort at the 26-year follow-up examination showed that obesity was linked to several cardiovascular diseases including coronary disease, congestive heart failure, and SCD (Hubert et al., 1983). Relative body weight was normalized to the 1959 Metropolitan Life insurance desirable weight tables, roughly corresponding to BMI of 22 kg/m^2^. When subjects weighing >130 or < 110% desired weight were compared over 26 years of follow-up, incidence of sudden death was 8 vs. 2.6% in men under 50 and 12 vs. 5.8% in men over 50 years old. The equivalent values for women were much lower at 2.4 vs. 0.5% in women under 50 and 4.1 vs. 0.6% in women over 50 years old.

A later analysis from 14,491 participants over 12 years of follow-up in the multiethnic ARIC study, waist-to-hip ratio and waist circumference, clinical measures of central adiposity, were measured in addition to BMI (Adabag et al., 2015). Waist-to-hip ratio had a stronger association with SCD than BMI or waist circumference in non-smokers, and this association was persistent even after adjustment for potential mediators such as DM, hypertension, lipid profile, coronary artery disease, heart failure, or left ventricular hypertrophy.

Although we are unaware of any clinical studies on the impact of bariatric surgery on SCD, one might predict that substantial weight loss would be protective. It is known that bariatric surgery normalizes the QT interval and QT dispersion, which should decrease risk of SCD (Russo et al., 2007; Mukerji et al., 2012).

Several studies also looked specifically at DM and risk of SCD. In one study, investigators analyzed data from 13,978 participants initially free of coronary disease in the ARIC study cohort (Kucharska-Newton et al., 2010), then examined the incidence of CVD events in subjects with or without DM at baseline with over 12 years of follow-up. The adjusted HRs were 3.77 for SCD, 3.78 for non-sudden cardiac death, and 3.20 for non-fatal myocardial infarction. DM was associated with both fatal and non-fatal CV events, and there was no specifically elevated risk for SCD. In a separate generational cohort, 10,594 Finnish subjects were followed for four decades (Eranti et al., 2016). As before, diabetic subjects had increased risk of both sudden, non-sudden cardiac death, and non-fatal cardiac events (HR 2.62, 3.05, and 3.63, respectively), but without an increased proportion of SCD.

Furthermore, higher levels of serum fatty acids and higher dietary saturated fat intake predict SCD (Oliver et al., 1968; Jouven et al., 2001; Chiuve et al., 2012). These observations suggest that the pro-arrhythmic effects of saturated fat on the heart may be nearly as important a risk factor for arrhythmia as obesity itself.

## Dyslipidemia, Inflammation, and Arrhythmia Development

**Figure 1:**
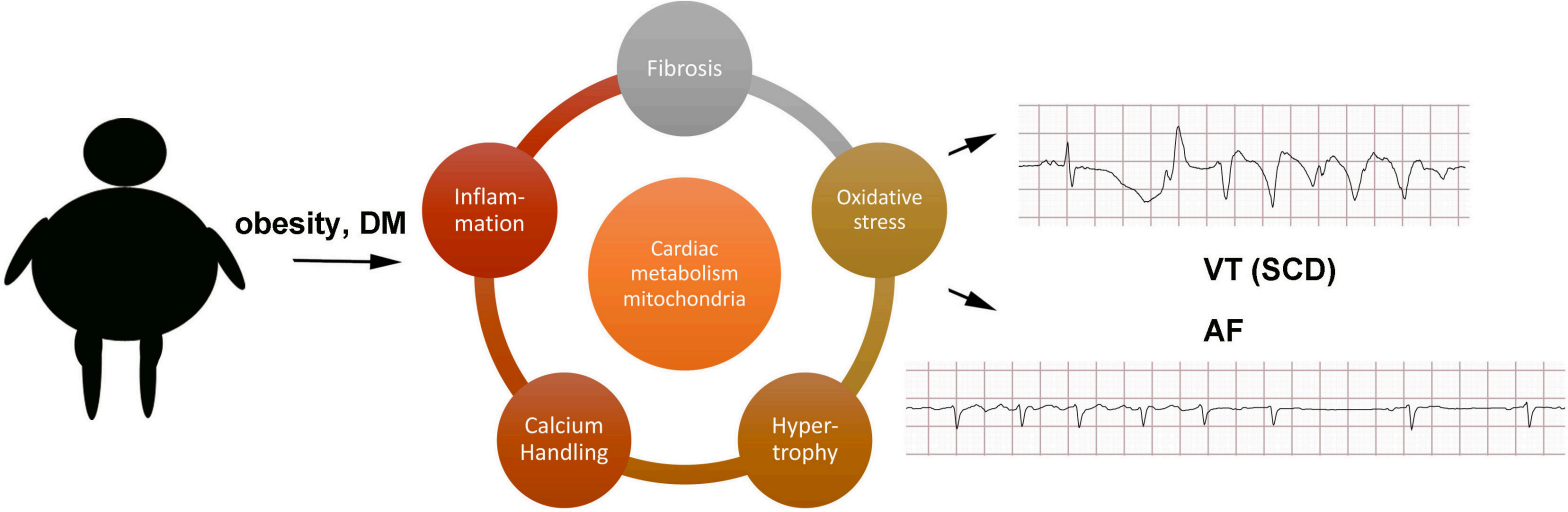
Schematic of possible mechanisms. AF, atrial fibrillation; DM, diabetes mellitus; SCD, sudden cardiac death; VT, ventricular tachycardia.

A full exploration of the molecular mechanisms causing arrhythmias in obesity and DM is beyond the scope of this review, and the interested reader should refer to other articles in this theme issue, but a few observations can be mentioned (Figure 1). Obesity is a state of chronic low-level inflammation, and AF patients have increased serum biomarkers of inflammation (Chung et al., 2001). Obesity promotes left ventricular hypertrophy even in the absence of hypertension (Cuspidi et al., 2014). Animal studies have shown that hypertrophy may be caused by activation of FOXO transcription factors and protein kinase D (Battiprolu et al., 2012; Huang et al., 2013).

Large animal models have demonstrated that obesity causes atrial lipid deposition, fibrosis, and inflammation (Abed et al., 2013b). More work is needed to determine which of these abnormalities is driving the pathophysiology of AF and which are epiphenomena. For example, in a sheep tachypacing model of AF, the aldosterone inhibitor eplerenone reduced atrial dilatation and fibrosis but did not prevent electrophysiologic remodeling (Takemoto et al., 2017). Inflammation could contribute to tissue remodeling and fibrosis. Cardiac fibrosis is increased in some obese humans, and there are small clinical trials showing that anti-fibrotic pharmacotherapy can improve cardiac function (Kosmala et al., 2013).

Cardiomyocytes from obese and diabetic patients have increased lipid accumulation, which is thought to contribute to the pathophysiology of heart failure and arrhythmia (Sharma et al., 2004; Lopaschuk et al., 2010). This syndrome of cardiac lipid overload has been termed lipotoxic cardiomyopathy (Wende and Abel, 2010). Transgenic models have demonstrated that cardiac lipid overload causes spontaneous arrhythmias (Morrow et al., 2011). The arrhythmogenic effect may be partly mediated by oxidative stress, which is increased in obesity and DM (Purohit et al., 2013; Samman Tahhan et al., 2017). Animal models of AF have mitochondrial damage in the atrial myocytes, and it seems likely that obesity could promote mitochondrial dysfunction (Wan et al., 2016). Mitochondrial antioxidants have been shown to reduce ventricular ectopy *in vivo* in a transgenic model of cardiac lipid overload (Joseph et al., 2016a). It has also been shown that cardiomyocyte lipid overload increases oxidative stress by activating the protein NOX2, which causes both mitochondrial dysfunction and abnormalities of internal calcium handling, promoting arrhythmia (Joseph et al., 2016b). Prior work using animal models has indicated that mitochondrial dysfunction is an important factor in ventricular arrhythmias caused by ischemia-reperfusion and non-ischemic heart failure (Akar et al., 2005; Xie et al., 2018). Similar mechanisms could be relevant to chronic conditions such as obesity, promoting AF and VT, but more work is needed to determine the molecular mechanisms.

## Future Directions

A large body of evidence indicates that there is a significant association between obesity and arrhythmia, and the evidence for an association with DM is somewhat mixed but still compelling. Structural cardiac factors such as left atrial size may mediate part of this effect, but cardiometabolic factors such as cardiac lipid overload and oxidative stress likely also play a role. There are also ethnic differences in the risk of AF beyond what can be explained by traditional risk factor analysis alone. Encouragingly, weight loss and dietary intervention can significantly reduce AF burden. Clinical trials at multiple centers are currently ongoing to address this question more directly.

Importantly, obese patients are at higher risk of ventricular arrhythmias leading to SCD, which may be related to impaired repolarization. Currently, no specific therapies to reduce the risk of SCD have identified, and this merits further study. It is possible that the newer medications for DM such as SGLT2 inhibitors or medications that modulate myocyte metabolism such as metformin may reduce the risk of arrhythmias, but more research is needed in this area. We would predict that medications that target cardiac lipid overload and/or mitochondrial dysfunction would be useful, if such agents could be developed with acceptable safety profiles.

## Author Contributions

EH and JM drafted and revised manuscript. MR and KH reviewed and edited manuscript.

### Conflict of Interest Statement

The authors declare that the research was conducted in the absence of any commercial or financial relationships that could be construed as a potential conflict of interest.

## Abbreviations

AF: atrial fibrillation
BMI: body mass index
CRF: cardiorespiratory fitness
CVD: cardiovascular disease
DM: diabetes mellitus
ECG: electrocardiogram
FHS: Framingham Heart Study
HbA1c: hemoglobin A1c
HR: hazard ratio
OR: odds ratio
OSA: obstructive sleep apnea
PUFA: polyunsaturated fatty acid
SCD: sudden cardiac death.
